# Cell Mechanotransduction With Piconewton Forces Applied by Optical Tweezers

**DOI:** 10.3389/fncel.2018.00130

**Published:** 2018-05-14

**Authors:** Fabio Falleroni, Vincent Torre, Dan Cojoc

**Affiliations:** ^1^Neuroscience Area, International School for Advanced Studies, Trieste, Italy; ^2^Cixi Institute of Biomedical Engineering, Ningbo Institute of Materials Technology and Engineering, Chinese Academy of Sciences, Zhejiang, China; ^3^Center of Systems Medicine, Chinese Academy of Medical Sciences, Suzhou Institute of Systems Medicine, Suzhou Industrial Park, Suzhou, China; ^4^Institute of Materials, National Research Council of Italy (CNR), Trieste, Italy

**Keywords:** cell mechanotransduction, calcium signaling, optical tweezers, cell indentation, piconewton forces

## Abstract

Mechanical stresses are always present in the cellular environment and mechanotransduction occurs in all cells. Although many experimental approaches have been developed to investigate mechanotransduction, the physical properties of the mechanical stimulus have yet to be accurately characterized. Here, we propose a mechanical stimulation method employing an oscillatory optical trap to apply piconewton forces perpendicularly to the cell membrane, for short instants. We show that this stimulation produces membrane indentation and induces cellular calcium transients in mouse neuroblastoma NG108-15 cells dependent of the stimulus strength and the number of force pulses.

## Introduction

Several sensory neurons transduce mechanical stimulations that provide the basis of hearing, touch, and noxious mechanical sensation (Ernstrom and Chalfie, 2002; Lumpkin et al., 2010). Mechanosensitive channels (Arnadóttir and Chalfie, 2010), however, are not found exclusively in these specialized neurons, but, rather, many other cells such as olfactory sensory neurons and possibly almost all neurons respond to an applied pressure (Connelly et al., 2015). Micropipettes/microneedles are used to pull and push cells and provide a localized mechanical stimulation (Hao and Delmas, 2011). Magnetic actuation of nanoparticles in combination with pressure-clamp electrophysiology have identified mechanically sensitive domains in mechanosensitive ionic channels (Wu et al., 2016). None of these methods, however, provide a precise and simultaneous measurement of the applied force and the indentation caused by the mechanical stimulus. The precise measurement of these two quantities is key in understanding the operation of the mechanical sensors and distinguishing between the membrane-tension model (Coste et al., 2010, 2015) and tether models (Sachs, 2015; Jin et al., 2017). In membrane-tension models, the change in membrane tension drives the opening of mechanosensitive channels, such as for Piezo 1 and 2 channels (Coste et al., 2012; Lewis and Grandl, 2015). In tether models, a link to the cytoskeleton controls channel gating, such as for the transient receptor potential (TRP) mechanosensitive channel (NOMPC) (Walker et al., 2000; Sachs, 2015; Jin et al., 2017). All of these channels have been reported to respond to membrane tension in the range of 0.1–10 mN/m (Zhang et al., 2015; Wu et al., 2017), and the pressure sensitivity of Piezo 1 and 2 mechanosensitive channels has been estimated to be in the range of some tens of mmHg (10^3^–10^4^) Pa (Charras et al., 2004; Coste et al., 2010; Wu et al., 2017). However, the applied force and pressure have not been measured precisely but only estimated.

It is possible to produce an indentation with nN forces to a cellular membrane by using a flexible cantilever in AFM (Gaub and Müller, 2017). In this case, the lowest force that can be exerted is limited by the thermal noise of the AFM cantilever, which in liquid is around 20 pN (Eghiaian and Schaap, 2011) limiting also the accuracy of the indentation measurement. To overcome these limitations, most of AFM experiments are routinely carried out from 0.1 to 100 nN (Lee et al., 2014; Gaub and Müller, 2017) and the indentation is performed at nN forces causing large deformations and possibly damages to the cell (Murphy et al., 2006). The force generated by growing microtubules (Dogterom and Yurke, 1997) or by f-actin–binding myosin motors (Finer et al., 1994) is in the order of 3–5 pN, therefore cells are likely to experience mechanical stimulations from just some pN up to several nN.

In order to exert controlled mechanical stimulations in the pN range, we established a method using an optical tweezers with a polystyrene microbead in an oscillatory optical trap. In this way it is possible to touch the cell in the vertical direction and to analyze cellular responses to forces in the range of 5–20 pN. By using this technique, we provide a method able to: (i) produce small (hundreds of nm) indentation of the cell membrane in the vertical direction; (ii) measure with nm precision the displacement of the microbead when the optical trap is set in contact with the cell membrane and determine precisely the applied force and the indentation produced into the cell membrane. Although the force exerted by the bead to the cell membrane is small, this stimulation is enough to trigger Ca^2+^ intracellular transients.

## Materials and methods

### Cell culture

Mouse neuroblastoma × rat glioma hybrid (NG108-15) cells were obtained from Sigma-Aldrich. The NG108-15 cells were cultured in Dulbecco's modified Eagle's medium (ThermoFisher) supplemented with 10% fetal bovine serum (FBS). The cells were cultured in a humidified incubator with 95% O_2_ and 5% CO_2_ at 37°C. For subculturing, the cells were washed with PBS and detached by minimal trypsinization (0.25% trypsin-EDTA solution) followed by incubation at 37°C until the cells detached. Fresh culture medium was added, and the cells were seeded in new culture flasks in a 1:4 ratio. For the experiments, cells were plated into coverslip coated with 50 μg/ml poly-L-ornithine (Sigma-Aldrich,) in 6 well plate culture containing Neurobasal medium (ThermoFisher) and with 2% B27 supplement (ThermoFisher) for 24–28 h to induce neuronal differentiation of NG108-15 cells.

### Calcium imaging

The cells were loaded with a cell-permeable calcium dye Fluo4-AM (Life Technologies) by incubating them with 4 μM Fluo4-AM dissolved in anhydrous DMSO (Sigma-Aldrich) and Pluronic F-127 20% solution in DMSO (Life Technologies) at a ratio of 1:1 in Krebs-Ringer's solution containing 119 mM NaCl, 2.5 mM KCl, 1 mM NaH_2_PO_4_, 2.5 mM CaCl_2_, 1.3 mM MgCl_2_, 11 mM D-glucose, and 20 mM HEPES (pH 7.4) at 37°C for 45 min. After incubation the cells were washed three times for at least 15 min total to allow complete intracellular de-esterification of the dye then transferred to the stage of an Olympus IX-81 inverted microscope equipped with LED illumination (X-Cite XLED1 from Excelitas Technologies). The experiments were performed at 37°C, and images were acquired using Micromanager software with an Apo-Fluor 60x/1.4 NA objective at a sampling rate of 5 Hz for 3–10 min. To avoid saturation of the signals, the excitation light intensity was attenuated by one neutral density filter (OD = 0.5, Thorlabs). Imaging experiments were conducted with Krebs-Ringer's solution containing 119 mM NaCl, 2.5 mM KCl, 1 mM NaH_2_PO_4_, 2.5 mM CaCl_2_, 1.3 mM MgCl_2_, 11 mM D-glucose, and 20 mM HEPES (pH 7.4).

### Mechanical cell stimulation using the oscillatory optical trap

**Figure 1:**
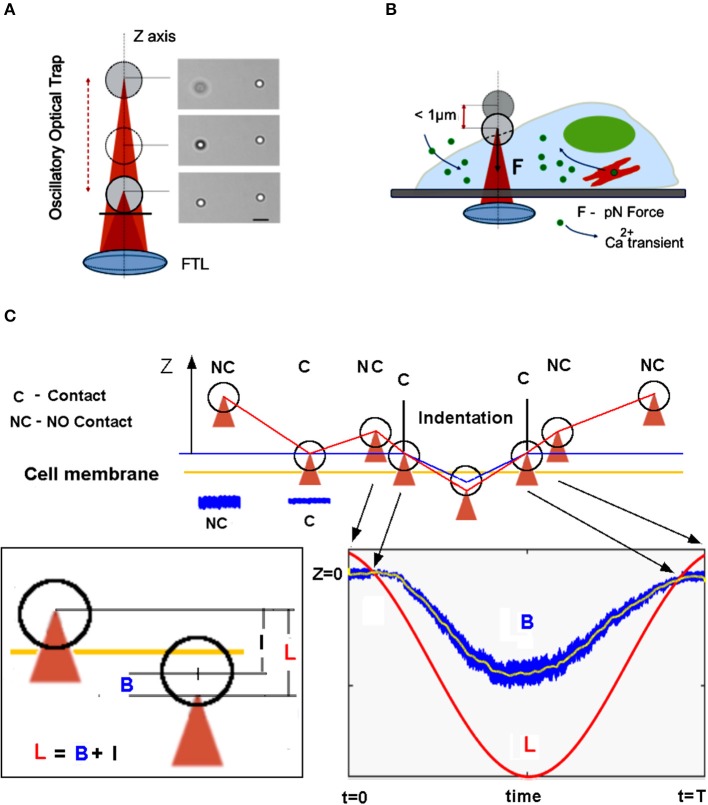
Overview of the mechanical stimulation with piconewton forces by optical tweezers. **(A)** The Oscillatory Optical Trap (OOT), implemented with a Focused Tunable Lens (FTL), enables continuous movement of the trap along the z-axis. Right side: image of a trapped bead shifted by 4 μm up from the focal plane; the bead on the right is fixed. Scale bar 5 μm. **(B)** Scheme of the mechanical stimulation inducing calcium transient experiment. **(C)** The measurement approach: the trap is lowered until the bead touches the cell membrane, indicated by the amplitude decrease of the bead fluctuations (see blue traces NC vs. C). The trap is moved up in NC position and the oscillatory movement of the trap begins to indent the cell membrane. The bead displacement, B from the center of the trap is measured (see blue trace in the bottom right inset). The relation between trap displacement L, bead displacement B, and indentation I is shown in the left inset.

**Figure 2:**
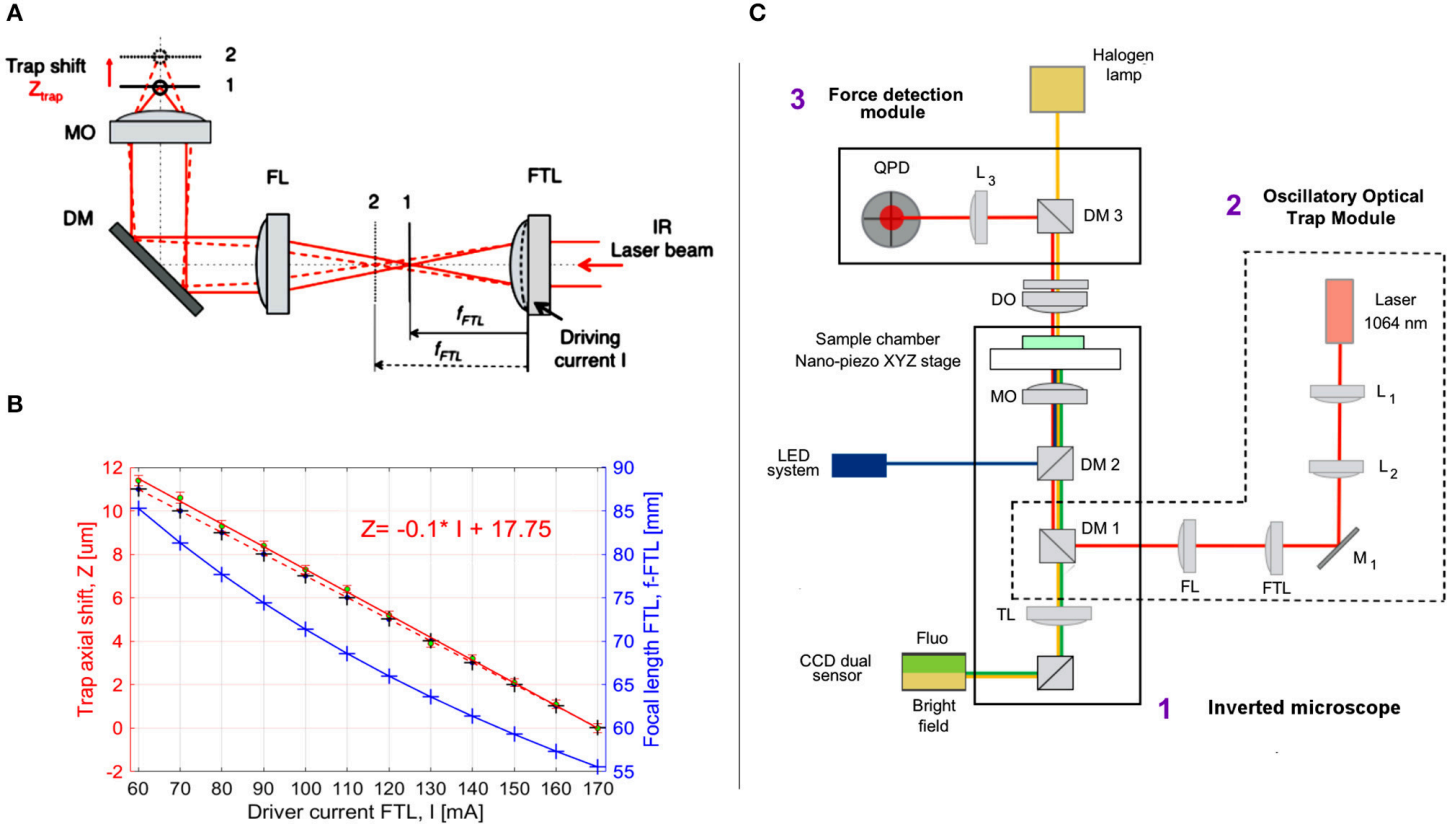
Optical manipulation and imaging setup. **(A)** Oscillatory Optical Trap (OOT): the collimated IR laser beam is focused by FTL in a focal point between planes 1 and 2, according to the driving current I. A convergent lens with fixed focal length (FL) re-collimates the laser beam, which is directed by the dichroic mirror (DM) into the microscope objective (MO), generating an optical trap between planes 1 and 2 near the MO focus. The trap is shifted axially in the range 0–12 μm from the nominal focal plane of the MO. **(B)** The trap shift, Z_trap_, as a function of the driving current I: Z_trap_ = Z_trap_(I). The blue crosses and curve represent the focal length of FTL as function of I; black crosses: theoretical values calculated for Z_trap_, linearly fitted with the red dotted line. The green dots represent the average of the Z_trap_ measured values (*n* = 5) linearly fitted with the red line: Z = −0.1044*I+17.75. **(C)** Optical manipulation and imaging setup: 1, inverted microscope; 2, oscillatory optical trap OOT; 3, Force measurement module. Optical components: L1, L2, convergent lenses, f1 = f2 = 100 mm; M1, mirror; FTL, Focus Tunable Lens, f_FTL_ = 55–90 mm; FL, Fixed focal Lens, f = 150 mm; DM1, dichroic mirror (900 dcsp, Chroma); DM2, dichroic mirror (XF22045, Chroma); TL, Tube Lens; MO, Microscope Objective, Olympus 60X, NA 1.4, oil immersion; DO, condenser objective, 10 X, NA 0.3; DM3, Dichroic Mirror (900dcsp, Chroma); L3, convergent lens, f = 40 mm; QPD, Quadrant Photo Diode.

To mechanically stimulate the cell, we used a polystyrene bead with a diameter d = 3.5-μm diameter (G. Kisker GbR,) optically manipulated in an oscillatory optical trap (OOT) (Figure 1 and Supplementary Video 1). The main component of the OOT is the Focused Tunable Lens (EL-10-30-NIR-LD, Optotune AG), of which focal length can be precisely tuned to change the vertical position of the trapped bead (Figure 2A). Cell stimulation is achieved by trapping the bead above the cell and then moving it against the cell membrane (Figure 1B).

**Figure 3:**
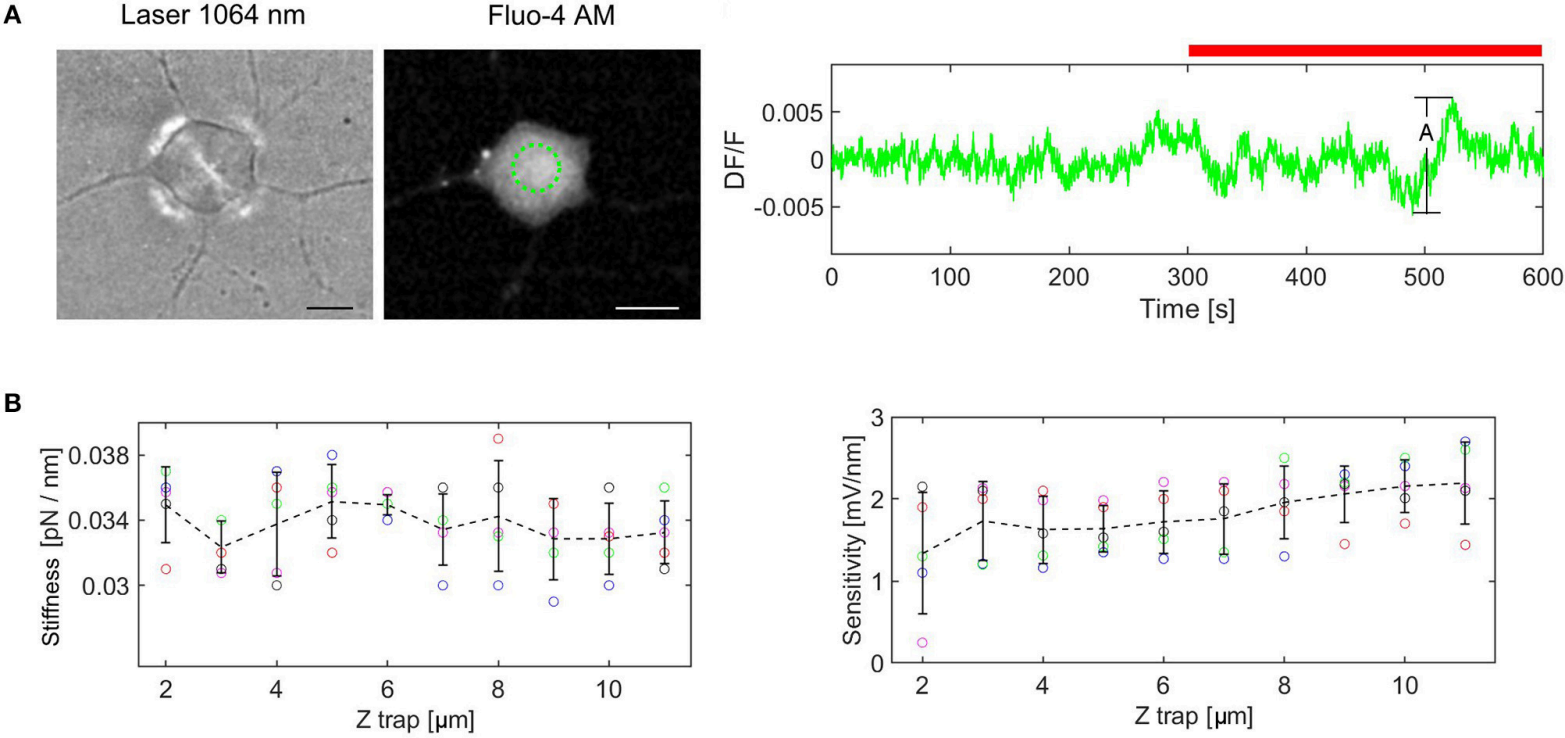
Control experiments. **(A)** Cell exposure to IR laser. The cell is exposed to laser beam (brightfield image at the left) and fluorescence is monitored for the ROI marked in green (image in the middle). DF/F measured for 10 min (red bar indicates laser irradiation), A = (DF/F)_max_, (DF/F)_min_ = 0.0125. **(B)** Trap stiffness and QPD sensitivity as a function of trap height. The error bars represent standard deviation (*n* = 5 experiments for each height). The dotted line links the mean values for each height.

To rule out the effect of the laser light on the cellular calcium transients we measured the fluorescence change (DF/F). DF/F taken over the cell, was measured for the cell not exposed to laser light (5 min) as reference, followed by cell exposed to laser (5 min). An example of the fluorescence change is shown in Figure 3A. The amplitude Ai, is defined as the difference between the maximum and minimum values of DF/F during the experiment. The experiment in Figure 3A displays the maximum amplitude, A = 0.0125 (*n* = 5 experiments). This value remains however well below the minimum value of the DF/F peaks, corresponding to Calcium transitions induced by force pulses (see **Figures 5**, **6**), indicating that the IR laser beam does not perturb the cell. The mean amplitude is 0.01 (*SD* = 0.0018) and this value is used to define the peak presence: A_p_ > 0.02, where A_p_ is the amplitude of the peak with respect to the baseline. Similar results, showing that the laser beam does not affect the cell, have been obtained also when a bead was trapped and kept above the cell.

The axial position of the trap could be regulated within a range of 0–12 μm above the focus of the microscope lens by changing the convergence of the beam entering the pupil of the lens (Figure 2). Beam convergence was changed using the focal length of the Focus Tunable Lens (EL-10-30-NIR-LD, Optotune AG), f_FTL_ = 55–90 mm in combination with a convergent lens of fixed focal length (FL), f_FL_ = 150 mm. The axial position of the trap from the focus of the microscope objective (trap shift) can be calculated by geometrical optics:

(1) $$\begin{array}{r} Z_{trap}=10^{3}\left[\frac{f_{MO}^{2}}{\frac{f_{FL}^{2}}{f_{FTL}+f_{FL}-d_{LT}}+d_{MO}-f_{FL}-f_{MO}}\right] \end{array}$$

where *Z*_trap_ is the trap axial shift in μm; *f*_*MO*_ is the focal length of the microscope objective, *f*_*MO*_ = 2 [mm]; *f*_*FL*_ is the focal length of the fixed lens, *f*_*FL*_ = 150 [mm]; *d*_*MO*_ is the distance between the fixed lens and the microscope objective in mm, *d*_*MO*_ = 380 [mm]; *d*_*LT*_ is the distance between the Focused Tunable Lens (FTL) and the fixed lens (FL) in mm, *d*_*LT*_ = 250 [mm]; *f*_*FTL*_ is the focal length of the FTL in mm, which is a function of the intensity current, *I* (in mA) applied to the FTL:

(2) $$\begin{array}{r} f_{FTL}=\frac{10^{3}}{\Phi }=\frac{10^{3}}{8.3+S•I} \end{array}$$

Φ is the power of the lens in diopters, 1 dpt = 1/m, *S* = 0.0571 dpt/mA is the FTL sensitivity (provided by the manufacturer). Introducing Equation (2) into Equation (1), one defines the axial trap shift, Z_trap_ as a function of the driving current, I. The focal length, f_FTL_ and the axial trap shift, Z_trap_ are plotted in Figure 2B for the driving current, I from 60 to 170 [mA]. Although f_FTL_ and Z_trap_ equations are not linear, for the limited range of I values represented in Figure 2B, Z_trap_ theoretical curve can be very well fitted with a line: Z_trap_ = −0.1^*^I + 17, (Root Mean Square Error RMSE = 0.021; *R*^2^ = 1). We then measured the experimental axial trap shift, ZE_trap_, assuming the position of the trap is in the focus of the beam and using the beam reflection by the coverslip (*n* = 5 measurements). The driving current I was first set to I = 170 mA and the nanopiezo (Nano-LPS100, Mad City Labs, Inc.) stage of the microscope moved vertically until the focus of the beam was observed (minimum spot on the coverslip). The current was then increased in steps of 10 mA and the stage moved until the focus was found again. The measured displacement of the stage represents the experimental value of the axial trap shift, *ZE*_*trap*_ (Figure 2B). These values are close to the theoretical values (MSE = 0.091), with larger differences observed for bigger axial trap shift values, due to the spherical aberrations (Theofanidou et al., 2004). Nevertheless, the linear fit of the experimental values is very good:

(3) $$\begin{array}{r} Z_{trap}=-0.1044•I+17.75 \end{array}$$

where RMSE = 0.121, R^2^ = 0.999, allowing to precisely control the trap position by the driving current. The coefficients p1 and p2 of the linear fit: *Z*_trap_ = p1^*^I + p2 were obtained with 95% confidence: p1 = −0.1044 (−0.1067, −0.1022); p2 = 17.75 (17.48, 18.02).

For mechanical stimulation, a bead is trapped above the cell at about 2–3 um (Figure 1C), then lowered toward the cell membrane in small steps (dI = 1–2 mA) until the variance of the axial displacement decreases considerably (>50%). This condition defines the criterium for the contact between the bead and the cell membrane. From this point, the bead is retracted back by one step (dI = 1 mA, corresponding to dZ = 100 nm), and then the trap oscillation is started. The maximum experimental error detecting the contact is thus given by the axial step dZ = 100 nm. The displacement of the bead, B from the center of the trap is however independent of this error, whereas the indentation of the cell, defined as: I = L-B is altered because of the error defining the starting point (NC in Figure 1C) for the oscillation, L. Therefore, the indentation I might be overestimated by 100 nm.

### Combined stimulation and imaging setup

The combined stimulation and imaging setup is based on an inverted microscope (Olympus IX81) and includes three main optical paths: IR optical trapping (Figure 2C, red line), brightfield imaging (yellow) and fluorescence imaging (blue/green). Two custom modules were adapted to the microscope: Oscillatory Optical Trap (OOT) and Force Detection (FD) to allow cell mechanical stimulation with forces measured in the range of 5–20 pN.

To direct the IR trapping beam toward the microscope lens (Olympus 60X, NA 1.4 oil immersion), we inserted a dichroic mirror (DM1 in Figure 2C) below the wheel of the fluorescence cubes, using a custom mounting that replaced the lens magnification adaptor of the Olympus microscope. The force exerted by the bead on the cell can be measured by the FD module using the IR laser light scattered by the trapped bead (probe) (Neuman and Block, 2004). To couple the FD module with the microscope optical path, the condenser lens of the microscope was replaced with a microscope lens (Olympus 10X, NA 0.3). This allows to suitably collect the IR light scattered by the probe (trapped bead) and project the interference pattern formed at the back-pupil plane onto the Quadrant Photo Detector (PDQ80A, Thorlabs).

The light used for fluorescence excitation (X-Cite XLED1, Excelitas Technology) was launched through the epifluorescence port at the back of the microscope. We used a CCD camera (Orca-D2, Hamamatsu) with a dual sensor to record the fluorescence image and the brightfield image simultaneously. This optical configuration enabled simultaneous optical trapping, cell mechanical stimulation, bright-field and epi-fluorescence imaging, and tracking the position of the trapped bead in X, Y, and Z directions. All the components (FTL, CCD camera, and QPD) were synchronized and controlled using a custom Labview software as well as the time-lapse control of the LED system. Data from the FTL and the QPD were acquired and digitized using a data acquisition board (NI PCI-6259, National Instruments).

### Force measurement

The displacement, S = (X, Y, Z) of the bead from the center of the trap can be measured with 0.2 ms time resolution and 5 nm precision using the Back-Plane Interferometry (BFI) technique (Neuman and Block, 2004).

**Figure 4:**
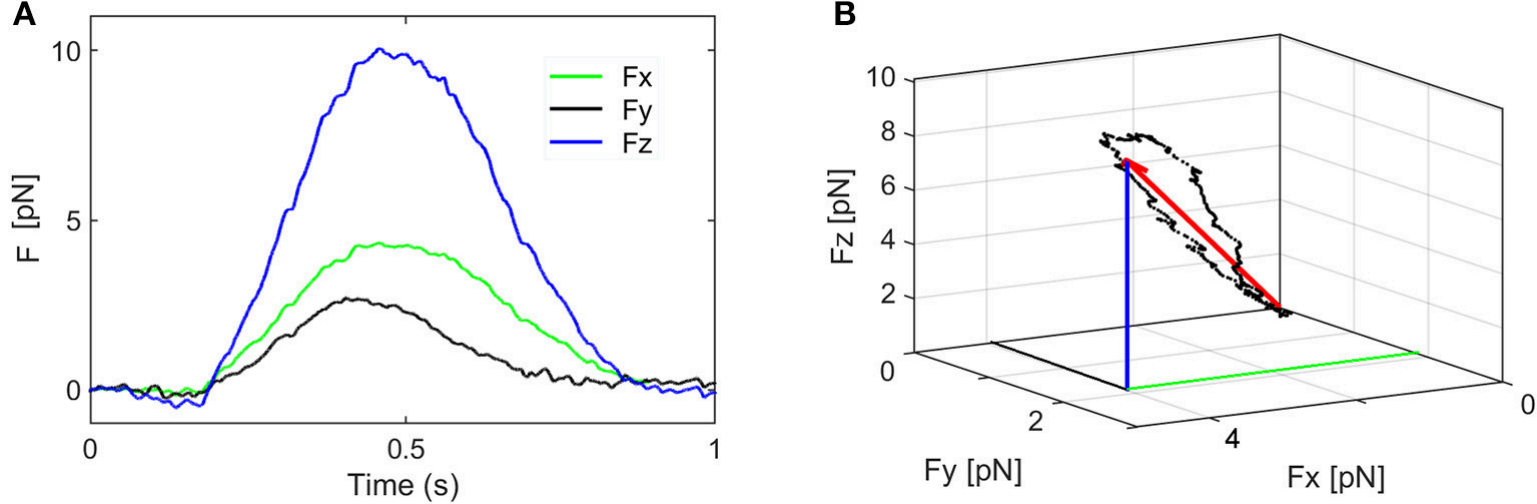
Components of the stimulation force. **(A)** The force components Fx, Fy, Fz, and their fluctuations during one stimulation period. **(B)** The orientation of the resultant force F and the change in its amplitude and direction (the coordinates of the black dots are given by Fx, Fy, Fz).

When the displacement, S, is less than 500 nm, it can be related to the force, F exerted on the bead (which is equal to the force exerted by the bead on the cell) by a proportionality factor, *k*:

(4) $$\begin{array}{r} F=F=k_{\;}•S \end{array}$$

where the force components are: *F* = (F_x_, F_y_, F_z_) and *k* = (k_x_, k_y_, k_z_) is called elastic constant or trap stiffness. The value of the force components Fx, Fy, Fz, are represented in Figure 4 for a stimulus period of 1 s. The orientation of the resultant force F and the change in its amplitude and direction are represented in Figure 4B and Supplementary Video 2. To keep the discussion simple, only the axial force component, Fz is considered here and the contribution of the smaller lateral forces is discussed later. To determine the trap stiffness, we recorded the bead fluctuations in the trap at a sampling frequency 5 kHz rate, for *t* = 5 s. The sampling frequency is much higher than the cutoff frequency of the constrained Brownian motion of the bead in the trap, thus allowing a correct sampling. Since the QPD signal is in Volts, this is converted in nm using the QPD sensitivity S_Z_ [mV/nm]. Both parameters, k_Z_ and S_Z_, can be determined using the power spectrum density (PSD) method (Neuman and Block, 2004). The PSD is calculated by Fourier transforming the QPD signal in Volts and is fitted with a Lorenz function to determine two constants: the plateau and the corner frequency, which define S_Z_ and k_Z_.

For a laser power of 25 mW at the sample plane, and the height of the trap, Z_trap_ = 6 μm from the focus of the objective, we obtained: k_Z_ = 0.035 [pN/nm] and S_*Z*_ = 1.9 [mV/nm]. When the height of the trap was altered, the bead remained trapped. However, since the convergence of the trapping laser changed slightly, we wondered how much this influenced the QPD signal. Therefore, we measured the trap stiffness and the detector sensitivity for different heights of the trap (from 2 to 11 μm) using a polystyrene bead (diameter d = 3.5 μm) in *n* = 5 different experiments. The results are plotted in the Figure 3B. The trap stiffness varies between a minimum value, kmin = 0.029 pN/nm to a maximum value, kmax = 0.039 pN/nm, with the mean value k = 0.034 pN/nm, (*SD* = 0.002). The sensitivity varies between 0.23 and 2.75 mV/nm, with a mean value of 1.817 mV/nm, (*SD* = 0.482). The sensitivity fluctuates more for Z_trap_ = 2–4 um, but it is much more stable for the region in which we are actually working (Z_trap_ = 4–8 um). Considering these results, the stiffness and sensitivity variations will generate maximum errors of 15 and 40%, respectively. If the errors are cumulative, the maximum error for force measurement would be 55%. However, for the height range we are working in the maximum error is reduced to 30%. Since the main goal of our paper is to show that calcium transients are induced by cell stimulation with forces of the order of 5–20 pN, i.e., much smaller (2–3 orders of magnitude) than the level of the forces previously reported (Lee et al., 2014; Gaub and Müller, 2017), the tolerance is acceptable in this context. Moreover, performing the calibration with the bead trapped above the cell before each indentation experiment, we could avoid this problem and regulate the stiffness to the nominal value by slightly adjusting the laser power.

### Immunohistochemistry

NG108-15 cells were fixed in 4% paraformaldehyde containing 0.15% picric acid in phosphate-buffered saline (PBS), saturated with 0.1 M glycine, permeabilized with 0.1% triton X-100, saturated with 0.5% BSA (all from Sigma- Aldrich) in PBS and the incubated for 1 h with primary antibodies: anti-Piezo1 (Alomone Labs). The secondary antibody was goat anti-rabbit Alexa Fluor 488 and the incubation time was 30 min. Nuclei were stained with 2 μg/ml in PBS Hoechst 33342 (Sigma-Aldrich) for 5 min. The cells were examined using a Nikon Eclipse C1si Confocal microscope. Images were acquired with a 40x 1.4 oil-immersion objective.

### Inhibition of mechanosensitive channels with GsMTx-4

GsMTx-4, a peptide toxin from *Grammostola spatulata* spider venom, was purchased from Tocris Bioscience and a 0.1 mM stock solution was prepared in distilled water. Working solutions were prepared by dilution in Krebs-Ringer's solution at the concentration of 8 μM. In pilot experiments, cells were treated with either GsMTx-4 or left untreated and used directly for calcium experiment. GsMTx4 is a gating modifier known for its selective inhibition of cation-permeable MCS channels belonging to the Piezo and TRP channel families.

### Data and statistical analysis

For calcium experiment the DF/F was quantified by custom developed code Matlab (MathWorks, Inc.) and Imagej software v1.6 (National Institutes of Health). The peaks of the Ca^2+^ transients were extracted using the threshold condition: A_p_ > 0.02, where A_p_ is the amplitude of the peak with respect to the baseline (Figure 3A). All the results are presented as mean ± *SD* and statistically differences were determined using a *t*-test, as appropriate with *p* < 0.05 considered statistically significant (GraphPad Prism 7, GraphPad software, San Diego, CA).

## Results

### Piconewton forces induce cell membrane indentation

**Figure 5:**
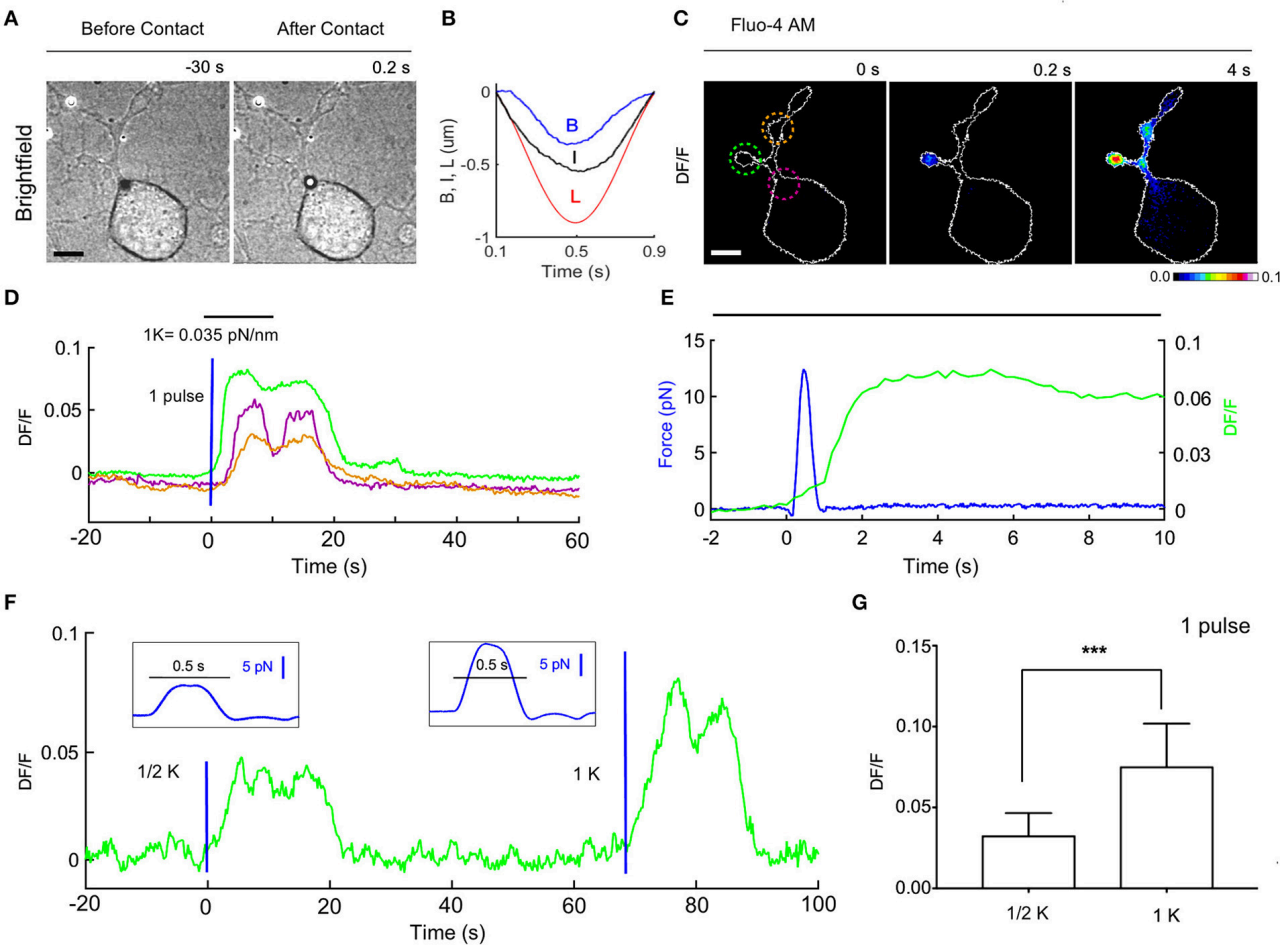
Ca^2+^ transients evoked by calibrated mechanical stimulations in NG10-15 cells. **(A)** Trapped bead above the cell, before (left) and after (right) contact with cell, scale bar: 10 μm. **(B)** Time course of the trap (L) and bead (B) measured displacements and calculated indentation I. **(C)** Fluorescence change, DF/F images, with 3 different ROIs to determine the fluorescence change vs. time **(D)**. **(E)** Detail of the time course of the applied force (blue) and the evoked calcium transient corresponding to green ROI. **(F)** Time course of evoked calcium transient (green) for an experiment in which a weak stimulus was first applied (trap stiffness, k/2), followed by a stronger stimulus (trap stiffness, k). The blue lines indicate application of the force pulses represented in inlets. **(G)** Statistics for the maximum of DF/F (mean ± *SD*) for k/2 (mean 0.0326 ± 0.004, *n* = 10) and k (0.075 ± 0.008, *n* = 12). *t*-test: ***P ≤ 0.001.

Cell membrane indentation is usually obtained with forces in the nN range (Gaub and Müller, 2017), much larger than inter-cellular forces which are in the pN range (Cojoc et al., 2007). By using an OOT with a micro-bead as the probe, we asked whether piconewton forces can induce a pressure large enough to indent the cell membrane, and how large can be this indentation. We trapped a polystyrene bead (diameter d = 3.5 μm) above a NG108-15 cell (Figure 5A) and adjusted the laser power so that the trap stiffness was k = 0.035 [pN/nm]. The bead was moved toward the cell membrane so to establish contact (Figure 1C). Following contact, the trapped bead was moved up by one step (100 nm) and then the trap was shifted down following a sinusoidal signal, L(t) with amplitude A = 1 μm and frequency f = 1 Hz. The interaction bead-cell membrane produces a displacement B(t) of the bead from the center of the trap and an indentation I(t) of the cell membrane, which are related to L(t) by the relation:

(5) $$\begin{array}{r} L\left(t\right)=\;B\left(t\right)+I\left(t\right)+Ct \end{array}$$

We measured the bead displacement *B(t)* from the center of the trap (see section Materials and Methods) and calculated *I(t)* = *L(t) – B(t)-Ct* (Figure 5B) and in the experiment illustrate in Figure 5. The maximum bead displacement is B_max_ = 350 nm, and the maximum indentation is I_max_ = 540. The time courses *B(t)* and *I(t)* show maxima at different time moments because the resistance opposed by the cell membrane to the bead pressure is different between pushing and pulling cycles. Using a trap stiffness, k_Z_ = 0.035 pN/nm we measured F_max_ = 12.3 pN. The pressure P produced by the vertical force is P = F/S, where S is the contact area between the bead and the cell, S = π·d·I. The maximum pressure P_max_ corresponds to the maximum force Fmax so that P_max_ = F_max_/S = 2.2 Pa = 0.017 mm Hg, which is 3 orders of magnitude less than the pressure exerted in the AFM stimulation using a bead of diameter d = 5 μm (Gaub and Müller, 2017). The variation of the membrane tension, produced by this pressure is: T = P^*^D/4, where D is the diameter of the contact circle: D = sqrt[I(d-I)] (Sachs, 2015). Using the above values, we obtain a change of tension: ΔT = 2.12 · 10^−3^ mN/m. This value is about 3 orders of magnitude smaller than the values previously assumed to trigger the opening of the mechanosensitive channels (Sachs, 2015; Jin et al., 2017). Since we measure also the lateral forces, Fx, Fy, we considered also their contribution: ΔTxy = Fxy/πD~ 1.6 ·10^−3^ mN/m, where Fxy = sqrt(F^2^x+ F^2^y) ~ 6 pN is the maximum lateral force, and D = 1.22 μm (I = 500 nm) is the contact circle diameter. The total tension change ΔT+ΔTxy = 3.62·10^−3^ mN/m is bigger but still much smaller than the values previously assumed to be necessary for the opening of mechanosensitive channels.

### A single force pulse induces Ca^2+^ transients in the cell

To evaluate whether forces in the pN range evoke a biological response we analyzed possible induced Ca^2+^ transients and we loaded the NG108-15 cells with the membrane permeable Calcium dye Fluo-4 (see section Materials and Methods). Before the mechanical stimulation was applied, fluorescence images were acquired at 5 Hz for 2 min to verify whether the intracellular Ca^2+^ level was stable and then we proceeded with the mechanical stimulation using the OOT. In the experiment illustrated in Figures 5A–E—with a maximum force equal to 12.3 pN and indentation equal to 540 nm-we observed an increase of intracellular Ca^2+^ level immediately after the stimulation (Figures 5C–E). This change was first localized in the neurite near the site of the mechanical stimulation, and then diffused into the other neurites (Figures 5C,D). The maximum fluorescence change (DF/F = 0.08) occurred in the first region about 5 s after stimulation, and with a delay of about 8 s in the other two regions. After about 20 s Ca^2+^ returned to the basal level. Similar changes of DF/F were observed in 12 experiments (DF/F peak: mean 0.075 ± 0.008 and Figure 5G) out of a total of 15 stimulated NG108-15 cells.

The mechanical forces exerted in experiments with trap stiffness k = 0.035 pN/nm (e.g., Figures 5A–E) had maximum values in the range 10–18 pN (mean 13.8 pN ± 2.5) and induced detectable changes of intracellular Ca^2+^. In order to establish a threshold for the mechanical stress which can induce Ca^2+^ intracellular transients, we decreased the trap stiffness k by a factor of 2, from k = 0.035 to k/2 = 0.0175 pN/nm. In this case, the maximum value of DF/F was 0.0326 ± 0.004 (*n* = 10), and no calcium activation was observed in 4 cells. The maximum forces were in the range 4–10 pN (mean 7.2 pN ± 1.5) which means the force applied was reduced by approximately the same factor as the trap stiffness. Considering also the values of the fluorescence change (DF/F—Figure 5G) our experiments show that the amplitude of Ca^2+^ transients scales with the applied force.

Our method allows a fast change of the trap stiffness, so it is possible to apply mechanical stimuli with different strengths to the same cell, as shown in Figure 5F.

### Adaptation to repetitive stimulations

**Figure 6:**
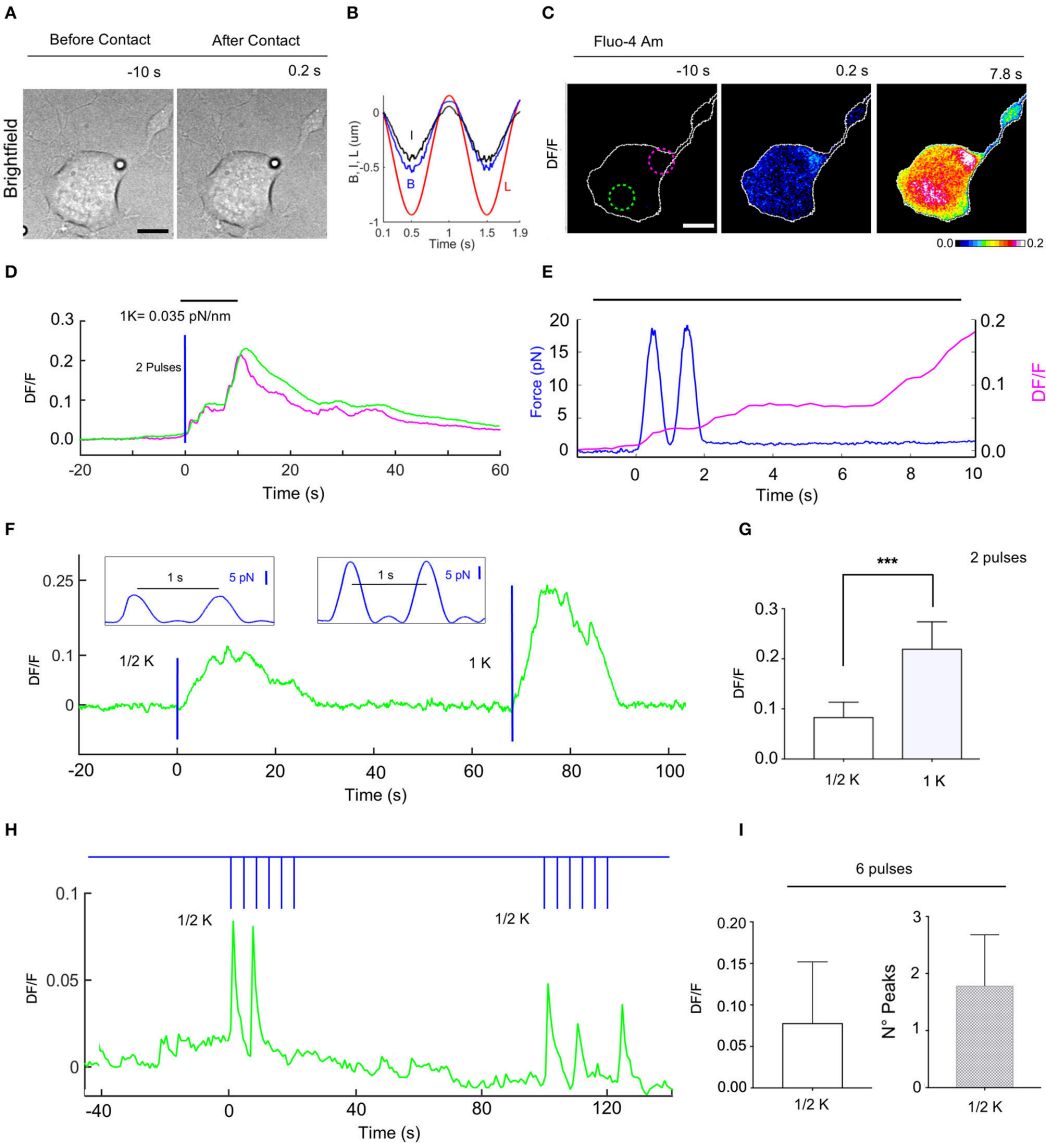
Calcium transients evoked by repetitive mechanical stimulations of NG108-15 cells. **(A)** Trapped bead above the cell, before (left) and after contact with cell (right), scale bar: 10 μm. **(B)** Time course of the trap (L) and bead (B) measured displacements and calculated indentation I for two pulses. **(C)** Fluorescence change, DF/F images, with 2 different ROIs to determine the fluorescence change vs. time **(D)**. **(E)** Time course of the applied force (blue trace) and the evoked calcium transient (magenta) from ROI. **(F)** Time course of evoked calcium transient (green) for an experiment in which a two pulses stimulus was first applied (trap stiffness, k/2), followed by a second two pulses stimulus (trap stiffness, k = 0.035 pN/nm). The blue lines indicate application of the stimuli with the force pulses represented in inlets. **(G)** Statistics for the maximum of DF/F values for k/2 (0.083 ± 0.011, *n* = 8) and k (0.22 ± 0.018, *n* = 9). *T*-test: ***P ≤ 0.001. **(H)** Time course of the evoked DF/F (green) and the mechanical stimulation (blue) composed of two train of 6 pulses at 0.25 Hz with k/2. **(I)** Statistics bar graph representing the mean value of DF/F induced by 6 pulses with k/2 (left, 0.078 ± 0.02, *n* = 13) and the mean value of the number of calcium peaks for each train of 6 pulses (right, 1.8 ± 0.2, *n* = 14).

We then applied mechanical stimulations composed of two consecutive force pulses with 1 Hz frequency (Figure 6) to observe whether cells show a cumulative force-dependent response to a pulsatile regime. Using a trap stiffness k = 0.035 pN/nm the maximum of the applied force (of two pulses) was 14.1 ± 2.5 pN (*n* = 9), and the amplitude of evoked Ca^2+^ transients was 0.22 ± 0.018 (Figure 6G), which was more than twice of that observed with one force pulse (0.075 ± 0.008, Figure 5G). Repeating the experiments with the trap stiffness k/2, the maximum of the applied force was 6.8 ± 2 pN (*n* = 8), and DF/F was 0.083 ± 0.011.

In order to explore the cell adaptation, we probed the response of NG108-15 cells to repetitive low strength (k/2) force pulses of 1 s with a resting time of 4 s (Supplementary Video 3 and Figure 6H). In these experiments, the DF/F had an amplitude of 0.078 ± 0.02 (*n* = 13) with a similar time (Figures 6H,I). However, in this case a DF/F peak could not be detected for every force pulse, a mean of 1.8 ± 0.2 (*n* = 14) pulses/train of pulses being detected (Figure 6I).

Although these gentle mechanical stimulations did not evoke any morphological change visible under bright-field illumination, when the mechanical stimulation was prolonged (1–3 min) the NG108-15 cell shrank, retracting the compartment submitted to low level mechanical stress by some microns (Supplementary Video 4).

### Expression of piezo1 channels in NG108-15 cells and MCS inhibition

**Figure 7:**
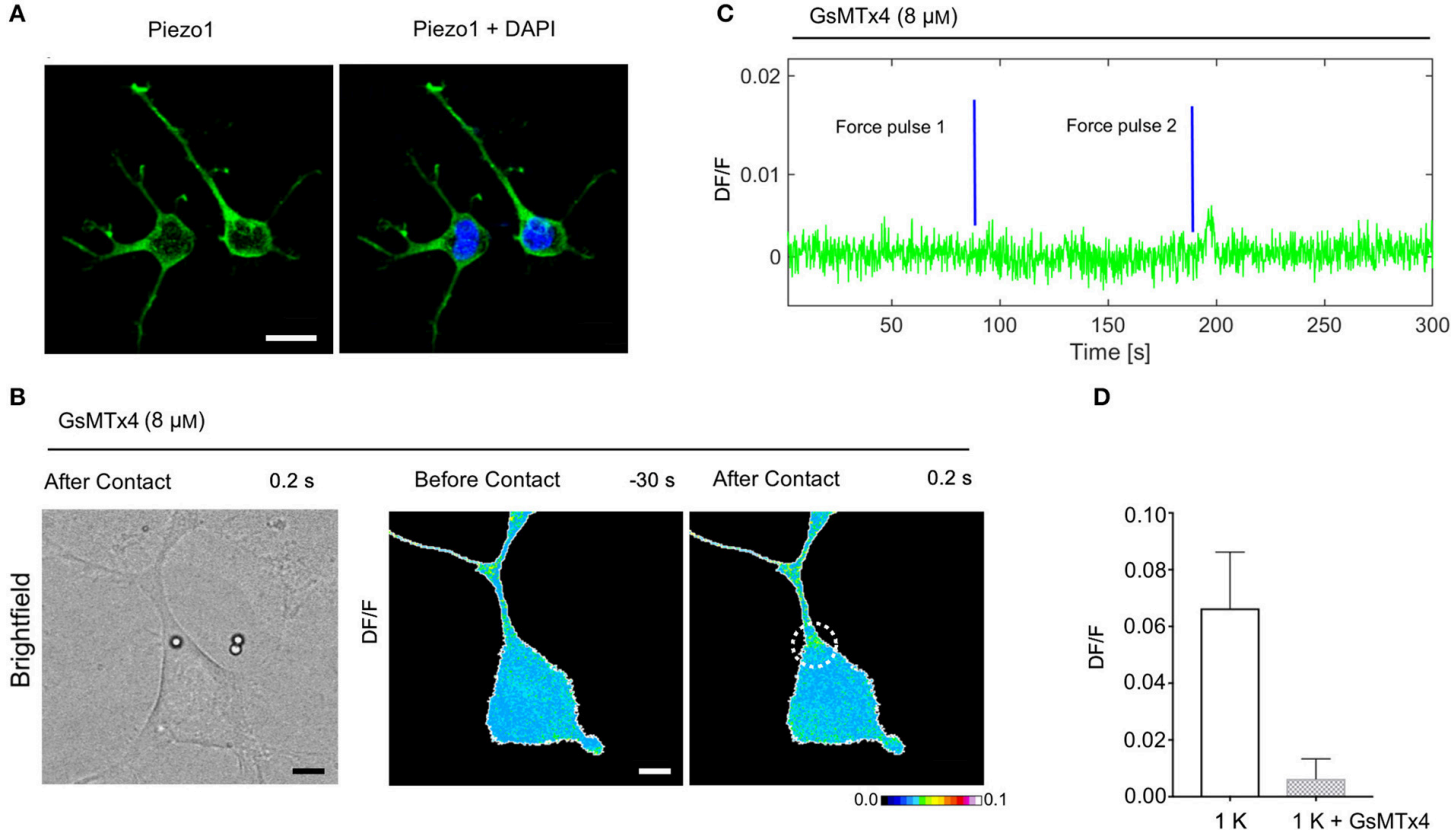
Expression of Piezo1 channels in NG108-15 cells and the effect of Gsmtx-4 on mechanically activated Ca^2+^ transient. **(A)** NG108-15 cells at 2DIV stained for Piezo 1 (green) and Hoechst 33342 nuclear stain (blue). **(B)** Brightfield and DF/F image obtained before and after the mechanical stimulation. **(C)** Time course of the evoked calcium transient (green trace) from white ROI in **(B)**. The blue lines indicate the application of the mechanical force. **(D)** Bar graphs represent fluorescence changes of Ca^2+^ (DF/F) in untreated cells stimulated with one pulse of strength k, as control group (*n* = 8) vs. cells treated with Gsmtx-4 (*n* = 10).

To examine if MCS channels are expressed in NG108-15 cells, we verified the presence of the PIEZO1 channel by immunostaining. The mechanosensitive channel Piezo1 is robustly expressed in the NG108-15 cells (Figure 7A) and is a good candidate for transducing the mechanical stimulus. Then, to identify better the source of the intracellular calcium elevation we tested the peptide GsMTx-4 that inhibits the cationic mechanosensitive channels (Gnanasambandam et al., 2017), as well as the Piezo channels (Bae et al., 2011). In the NG108-15 cells, we observed that the Gsmtx-4 at the concentration of 8 μM inhibited the occurrence of Ca2+ transient almost completely: in the presence of GsMTx-4 the amplitude of Ca^2+^ transient DF/ was 0.006 ± 0.002 (*n* = 10), compared with what obversed from the untreated cells during the same experimental session 0.067 ± 0.007 (*n* = 8; Figures 7B–D).

## Discussion

We have developed an optical tweezers method to apply weak forces in the 5–20 pN range to the cell membrane and demonstrated that these small forces produce an indentation of the cell membrane and trigger Ca2+ transients in NG108-15 cells. A similar approach, but with a fixed trap and moving the piezostage, has been recently used to investigate the indentation in breast cancer cells (Coceano et al., 2015; Yousafzai et al., 2016). Our approach, using an oscillatory optical trap (OOT) allows to keep the cell in focus during the stimulation, enabling optimum brightfield, and fluorescence imaging of the cell. This unique feature is possible by using the Focused Tunable Lens (FTL), which is positioned in an optical path separated from the imaging optical path of the microscope. Another possibility to decouple sample imaging from the trapping position has been reported using spatial light modulators (Emiliani et al., 2005) but this technique is more complex, less precise, and slower than the OOT with FTL.

It is known, that when the mechanical stress is applied, an early increase in intracellular calcium is generated (Godin et al., 2007), possibly caused by the opening of mechanosensitive channels which can be followed by larger calcium waves likely due to the release of calcium from internal stores such as the endoplasmatic reticulum and/or the delayed opening of additional calcium-permeable ionic channels (Wang et al., 2005; Kim et al., 2015). We found that localized mechanical stress induces a Ca2+ elevation immediately after stimulation and nearby the site where the stimulation was applied (Figures 5C, 6C). Interestingly, the amplitude of the Ca2+ oscillations for two pulses (strength k, force 14.1 pN ± 2.5) reached 0.22 ± 0.018 vs. the amplitude corresponding to one pulse (strength k, force 13.8 pN ± 2.5) stimulation: 0.075 ± 0.008 (Figures 5G, 6G). These results suggest that mechanical stimulation can modulate the calcium signal transduction pathway.

We also showed that the treatment of the NG108-15 cells with GsMTx4 to specifically inhibit mechanically activated cation channels, strongly reduced the calcium response upon the mechanical stress. This suggests that the mechanosensitive ion channels are necessary for the calcium mechanotransduction. Moreover, when low regime mechanical stimulation was prolonged (repeated trains of weak pulses, k/2) NG108-15 cells retract the compartment under the mechanical stress; these results are in agreement with the previous observation in which calcium influx trough mechanosensitive channels can induce retraction (Doyle et al., 2004) and inhibits neurite outgrowth in opposition to other influx pathways and releases from the intracellular store (Jacques-Fricke et al., 2006).

Our work has two major implications: first, we have shown how to apply mechanical stimuli under controlled conditions, the force and indentation of which are measured directly and precisely; second, in addition to mechanotransduction operating for large forces in the range of 0.1–500 nN, we have shown that very low levels of mechanical stress (5–20 pN) are able to induce a calcium intracellular response in NG108-15.

Our results suggest that the mechanotransduction pathway may be sensitive to physiologically mechanical touches, characterized by pN forces, as the one produced by a moving lamellipodium (Cojoc et al., 2007). Understanding the molecular and biophysical mechanism of how cells locally regulate the complex mechanical response may clarify how cells change shape and control their migratory behavior. Therefore, mechanical signaling among cells is important and ubiquitous but still needs to be better clarified.

## Author contributions

FF, DC, and VT designed the study. FF and DC designed and implemented the experimental setup. FF performed experiments. FF and DC collected and analyzed data. FF, DC, and VT wrote the manuscript.

### Conflict of interest statement

The authors declare that the research was conducted in the absence of any commercial or financial relationships that could be construed as a potential conflict of interest.
