# Corrigendum: Cell Mechanotransduction With Piconewton Forces Applied by Optical Tweezers

**DOI:** 10.3389/fncel.2018.00180

**Published:** 2018-06-20

**Authors:** Fabio Falleroni, Vincent Torre, Dan Cojoc

**Affiliations:** ^1^Neuroscience Area, International School for Advanced Studies, Trieste, Italy; ^2^Cixi Institute of Biomedical Engineering, Ningbo Institute of Materials Technology and Engineering, Chinese Academy of Sciences, Zhejiang, China; ^3^Center of Systems Medicine, Chinese Academy of Medical Sciences, Suzhou Institute of Systems Medicine, Suzhou Industrial Park, Suzhou, China; ^4^Institute of Materials, National Research Council of Italy (CNR), Trieste, Italy

**Keywords:** cell mechanotransduction, calcium signaling, optical tweezers, cell indentation, piconewton forces

We noticed that two of Vincent Torre's affiliations were missing:

“Cixi Institute of Biomedical Engineering, Ningbo Institute of Materials Technology and Engineering, Chinese Academy of Sciences, Zhejiang, China; Center of Systems Medicine, Chinese Academy of Medical Sciences, Suzhou Institute of Systems Medicine, Suzhou Industrial Park, Suzhou, China.”

Furthermore, there are two minor errors in the text:

A correction has been made to Results, “Expression of Piezo1 Channels in NG108-15 Cells and MCS Inhibition”:

“Ca2+ transient almost completely: in the presence of GsMTx-4 the amplitude of Ca2+ transient DF/”

has been changed to:

“Ca^2+^ transient almost completely: in the presence of GsMTx-4 the amplitude of Ca^2+^ transient DF/F”.

In “Discussion paragraphs 1 and 2,” Ca2+ has been changed to Ca^2+^.

None of these irregularities affect the original meaning of the article.

The original article has been updated.

## Conflict of interest statement

The authors declare that the research was conducted in the absence of any commercial or financial relationships that could be construed as a potential conflict of interest.

